# Occupational Ladder Fall Injuries — United States, 2011

**Published:** 2014-04-25

**Authors:** Christina M. Socias, Cammie K. Chaumont Menéndez, James W. Collins, Peter Simeonov

**Affiliations:** 1 EIS officer, CDC; 2 Division of Safety Research, National Institute for Occupational Safety and Health, CDC

Falls remain a leading cause of unintentional injury mortality nationwide, and 43% of fatal falls in the last decade have involved a ladder (1). Among workers, approximately 20% of fall injuries involve ladders (2–4). Among construction workers, an estimated 81% of fall injuries treated in U.S. emergency departments (EDs) involve a ladder (5). To fully characterize fatal and nonfatal injuries associated with ladder falls among workers in the United States, CDC’s National Institute for Occupational Safety and Health (NIOSH) analyzed data across multiple surveillance systems: 1) the Census of Fatal Occupational Injuries (CFOI), 2) the Survey of Occupational Injuries and Illnesses (SOII), and 3) the National Electronic Injury Surveillance System–occupational supplement (NEISS-Work). In 2011, work-related ladder fall injuries (LFIs) resulted in 113 fatalities (0.09 per 100,000 full-time equivalent* [FTE] workers), an estimated 15,460 nonfatal injuries reported by employers that involved ≥1 days away from work (DAFW), and an estimated 34,000 nonfatal injuries treated in EDs. Rates for nonfatal, work-related, ED-treated LFIs were higher (2.6 per 10,000 FTE) than those for such injuries reported by employers (1.2 per 10,000 FTE). LFIs represent a substantial public health burden of preventable injuries for workers. Because falls are the leading cause of work-related injuries and deaths in construction, NIOSH, the Occupational Safety and Health Administration, and the Center for Construction Research and Training are promoting a national campaign to prevent workplace falls (2). NIOSH is also developing innovative technologies to complement safe ladder use (6).

The Bureau of Labor Statistics (BLS) administers the CFOI† each year to enumerate all fatal occupational injuries using multiple data sources. BLS also implements the annual SOII§ to estimate injury and illness involving ≥1 DAFW from a nationally representative sample of employer-collected records. The NEISS-Work¶ surveillance system estimates work-related injuries treated annually in EDs. LFI cases were identified using the Occupational Injury and Illness Classification System,** where the injury source was a ladder and the injury event was a fall to a lower level.

To calculate rates, labor force denominator estimates from the U.S. Current Population Survey (CPS)†† for workers aged >15 years were used. Confidence intervals for NEISS-Work estimates accounted for the variance arising from the stratified cluster sample. The number, percentage, and rate of LFIs from CFOI, SOII, and NEISS-Work in 2011 were compared across demographic, work, and injury characteristics where available (Table).

Men and Hispanics had higher rates of fatal and nonfatal LFIs compared with women and non-Hispanic whites and persons of other races/ethnicities (Table). LFI rates increased with age, except for injuries treated in EDs. Fatality rates were substantially higher for self-employed workers (0.30 per 100,000 FTE workers) than salary/wage workers (0.06 per 100,000 FTE workers). Establishments with the fewest employees had the highest fatality rates. The construction industry had the highest LFI rates compared with all other industries. Across all industries, the highest fatal and nonfatal LFI rates were in the following two occupation groups: construction and extraction (e.g., mining) occupations, followed by installation, maintenance, and repair occupations. Head injuries were implicated in about half of fatal injuries (49%), whereas most nonfatal injuries involved the upper and lower extremities for employer-reported and ED-treated nonfatal injuries.

Severity of nonfatal LFIs was assessed using median DAFW (for employer-reported injuries) and disposition after ED treatment. Those with the highest median DAFW included men (21 days), workers aged 45–54 years (25 days), Hispanics (38 days), and construction and extraction workers (42 days). Workers with lower extremity (22 days) and multiple body part (28 days) injuries had higher median DAFW compared with other injuries. The hospital admission rate for ED-treated LFIs was 14%, almost three times the estimated overall hospital admission rate of 5% in the NEISS-Work survey for 2011, suggesting that LFIs were more severe compared with all other ED-treated injuries.

Fall height was documented for 82 of 113 fatalities and an estimated 11,400 of 34,000 nonfatal ED-treated LFIs (Figure). For nonfatal LFIs, nearly 90% were from heights <16 feet (<4.9 m) and fall heights of 6–10 feet (1.8–3.0 m) were most common, accounting for 50% of ED-treated LFIs. For fatal LFIs, fall heights of 6–10 feet (1.8–3.0 m) were most common but accounted for only 28% of all fatalities.

## Discussion

Falls, particularly falls from ladders, contribute substantially to injuries in the workplace. To gain a comprehensive picture of the injury burden caused by ladder falls at work, cases from three different occupational surveillance systems were examined. Each system offers a different perspective on injuries. Current literature on LFIs indicates a higher burden of injuries to men, older workers, and construction workers (4,5). Although this analysis found similar results, it also indicated that Hispanics, self-employed workers, and workers in smaller establishments had disproportionately higher LFI rates. Higher rates of LFIs were identified in installation, maintenance, and repair occupations, in addition to construction and extraction workers. This report adds to the literature on occupational fall injuries by providing a comprehensive, multisystem view of LFIs across all occupational groups using the most recent surveillance data available in the United States. This analysis provides a baseline to the multiagency falls prevention campaign that started in 2012 (2).

What is already known on this topic?

Falls remain a leading cause of injury in the general population and among workers, particularly construction workers. Ladders contribute substantially to the public health burden of fall injuries, but most research in this area focuses on construction workers.

What is added by this report?

Analysis of data from three surveillance systems showed that in 2011, work-related ladder fall injuries (LFIs) resulted in 113 fatalities, an estimated 15,460 nonfatal injuries that involved ≥1 days away from work, and an estimated 34,000 nonfatal injuries treated in emergency departments. Workers who are male, Hispanic, older, self-employed, work in smaller establishments, and work doing construction and extraction or installation, maintenance, and repair experience higher LFI rates.

What are the implications for public health practice?

The findings of this study reinforce the need for workplace safety research to prevent falls, including developing and disseminating innovative technologies to prevent LFIs. Employers, health-care providers, and safety professionals should collaborate to ensure availability and training of safe ladder practices.

The findings of this report are subject to at least four limitations. First, inclusion of cases is dependent on identifying the relationship between injury and work, which is not always clear, particularly for nonfatal injuries. Second, it is well recognized that nonfatal injury surveillance systems are subject to reporting and recording biases, which might result in underestimations of injury counts and rates (7). For example, not all demographic characteristics are pertinent to medical treatment, and therefore might be underreported during ED treatment. Such biases were minimal for CFOI because it is a census, rather than a sampled survey. Third, in this analysis, all workers were included in the denominator to calculate rates, which might underestimate the injury burden. A preferable denominator to understand LFI risk would be workers who used ladders in 2011, which might be available in future studies (8). Finally, this study is unable to evaluate adherence to safety recommendations.

Injuries from ladder falls can be severe but are preventable. Medical professionals might recommend safe ladder practices to their patients, such as those published by the American Academy of Orthopedic Surgeons as part of the Prevent Injuries Campaign (9). To prevent ladder falls, employers should consider the following steps: 1) plan the work to reduce or eliminate the need for using ladders by applying safety-in-design and constructability principles to finish as much of the work as possible on the ground; 2) provide alternative, safer equipment for extended work at elevation, such as aerial lifts, supported scaffolds, or mast climbing work platforms; 3) provide properly selected and thoroughly inspected ladders, that are well-matched to employee weight, task, and location; 4) when applicable, provide proper accessories to supplement safe ladder use; and 5) provide adequate ladder safety information and training for employees (6,9). Familiarity and compliance with the provisions of safety regulations, such as recognizing ladder types and conditions, and using ladder positioning and other safe ladder practices, are crucial to reducing injuries from ladder falls (2).

NIOSH safety research in this area focuses on innovative technologies to improve safe ladder use (6). For example, NIOSH recently developed and released a smartphone application (app) “Ladder Safety” (available at http://www.cdc.gov/niosh/topics/falls), which provides graphic-oriented, interactive, and easy-to-use reference materials, safety guidelines, and checklists for extension ladder selection, inspection, and use. The app is a convenient ladder safety performance and training tool and is available as a free download for Apple and Android mobile devices in English and Spanish (10).

## Figures and Tables

**FIGURE:**
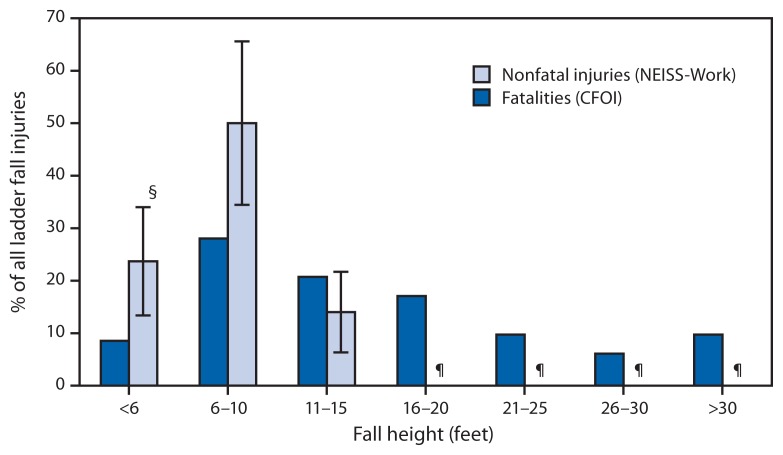
Percentage of ladder fall fatalities^*^ and nonfatal ladder fall injuries treated in emergency departments,^†^ by fall height (when documented) — United States, 2011 **Abbreviations:** CFOI = Census of Fatal Occupational Injuries; NEISS-Work = National Electronic Injury Surveillance System–occupational supplement; BLS = Bureau of Labor Statistics. ^*^ Percentage of ladder fall fatalities were generated with restricted access to BLS CFOI microdata and might differ from results released by BLS. Fatality counts on which the percentages are calculated are based are 82 cases where ladder height was indicated and include deaths to workers of all ages, volunteer workers, and resident military personnel. ^†^ Excludes 31 fatalities and 22,600 nonfatal injuries with unknown fall height. ^§^ 95% confidence interval. ^¶^ Nonfatal emergency department–treated injuries in this height category did not meet criteria for publication without compromise of confidentiality.

**TABLE:** Number, percentage, and rate of fatal and nonfatal occupational ladder fall injuries, by selected characteristics and data source — United States, 2011

Characteristic	CFOI (fatalities)			SOII (nonfatal injuries reported by employers)						NEISS-Work (nonfatal injuries treated in EDs)				
		
	No.	%*	Rate†	No.	(95% CI)	%*	Rate§	(95% CI)	Median DAFW¶	No.	(95% CI)	%*	Rate**	(95% CI)
**Total**	**113**	**100**	**0.09**	**15,460**	**(±550)**	**100**	**1.2**	**(±0.1)**	**20**	**34,000**	**(±6,800)**	**100**	**2.6**	**(±0.5)**
**Sex**														
Men	—††	—	—	12,510	(±470)	81	1.7	(±0.1)	21	30,100	(±6,300)	89	4	(±0.2)
Women	—	—	—	2,940	(±220)	19	0.5	(±0.1)	13	3,900	(±1,300)	11	0.7	(±0.2)
**Age group (yrs)**														
20–34	15	13	0.04	3,990	(±680)	26	1	(±0.2)	—	11,000	(±2,500)	32	2.7	(±0.5)
35–44	16	14	0.05	3,370	(±240)	22	1.1	(±0.1)	12	9,900	(±2,500)	29	3.3	(±0.6)
45–54	31	27	0.09	4,020	(±260)	26	1.2	(±0.1)	25	7,100	(±2,500)	21	2.2	(±0.5)
55–64	33	29	0.16	3,180	(±230)	21	1.5	(±0.2)	17	4,400	(±1,500)	13	2.1	(±0.5)
≥65	18	16	0.35	—		—	—		—	—		—	—	
**Race/Ethnicity** §§														
White, non-Hispanic	76	67	0.08	6,670	(±330)	43	0.7	(±0.1)	16	19,900	(±6,100)	59	2.2	(±0.4)
Other, non-Hispanic	8	7	0.04	—		—	—		—	2,000	(±1,000)	6	0.9	(±0.5)
Hispanic	29	26	0.15	2,460	(±200)	16	1.3	(±0.2)	38	5,800	(±2,800)	17	3.1	(±1.3)
Unknown	—	—	—	5,440	(±300)	35	—		15	—		—	—	
**Employment status**														
Employed (salary/wage)	73	65	0.06							27,800	(±5,600)	82	2.4	(±0.1)
Self-employed/farm/family business/other	40	35	0.30							3,800	(±4,000)	11	2.8	(±1.1)
**Establishment size** ¶¶														
1–19 employees	70	62	0.35											
20–99 employees	12	11	0.06											
≥100 employees	10	9	0.01											
**Industry** *** †††														
Agriculture/forestry/fishing	—	—	—	—		—	—		—	—		—	—	
Mining	—	—	—	—		—	—		—	—		—	—	
Construction	64	57	0.74	3,600	(±390)	23	7.4		40	11,500	(±3,000)	34	13	(±2.5)
Manufacturing	9	8	0.06	1,160	(±110)	8	1.0		15	—		—	—	
Trade	—	—	—	2,770	(±220)	18	—		—	4,500	(±1,300)	13	2.5	(±0.6)
Transport/warehouse/utilities	—	—	—	—		—	—		—	—		—	—	
Services (excluding health care)	27	24	0.04	4,400	(±380)	29	—		—	5,200	(±1,600)	15	0.8	(±0.2)
Health care/social services	—	—	—	—		—	—		—	—		—	—	
**Occupation** §§§														
Management/business/finance	—	—	—	—		—	—		—	—		—	—	
Professional and related	—	—	—	—		—	—		—	—		—	—	
Service	14	12	0.06	2,520	(±370)	16	1	(±0.2)	—	1,900	(±700)	6	0.8	(±0.3)
Sales and related	—	—	—	1,390	(±150)	9	1	(±0.2)	17	2,200	(±1,000)	7	1.6	(±0.7)
Office/administrative support	—	—	—	—		—	—		—	—		—	—	
Farming/fishing/forestry	—	—	—	—		—	—		—	—		—	—	
Construction/extraction	57	50	0.83	3,510	(±240)	23	5.1	(±0.4)	42	10,700	(±2,900)	32	16	(±2.9)
Installation/maintenance/repair	17	15	0.34	3,650	(±250)	24	7.3	(±0.6)	28	2,900	(±1,500)	9	5.7	(±2.6)
Production	8	7	0.10	—		—	—		—	—		—	—	
Transport/material moving	5	4	0.06	—		—	—		—	—		—	—	
**Part of body injured** ¶¶¶														
Head	55	49	0.04	810	(±120)	5	0.1	(±0.1)	10	4,900	(±1,500)	14	0.4	(±0.1)
Trunk (chest/back/abdomen)	13	12	0.01	2,790	(±210)	18	0.2	(±0.1)	13	8,300	(±2,200)	24	0.6	(±0.1)
Upper extremities	—	—	—	3,280	(±230)	21	0.3	(±0.1)	15	9,400	(±2,400)	28	0.7	(±0.1)
Lower extremities	—	—	—	4,960	(±290)	32	0.4	(±0.1)	22	10,000	(±2,700)	29	0.8	(±0.1)
Multiple body parts	40	35	0.03	3,550	(±240)	23	0.3	(±0.1)	28	—		—	—	
**Disposition**														
Treated and released										29,200	(±6,000)	86	2.2	(±0.1)
Admitted										4,800	(±1,800)	14	0.4	(±0.1)

**Abbreviations:** CFOI = Census of Fatal Occupational Injuries; SOII = Survey of Occupational Injuries and Illnesses; NEISS-Work = National Electronic Injury Surveillance System–occupational supplement; ED = emergency department; CI = confidence interval; DAFW = days away from work; FTE = full-time equivalent; BLS = Bureau of Labor Statistics; RSE = relative standard error; NAICS = North American Industry Classification System; SOC = Standard Occupational Classification.

* Percentages might not sum to 100 because of exclusions and rounding.

† Per 100,000 FTE (FTE = 2,000 hours worked per year) per BLS publication requirements. Numbers of deaths are reported for workers of all ages, whereas rates are for workers aged ≥16 years. Rates were calculated by CDC based on the number of fatalities from restricted data from the BLS CFOI during 2011 and might differ from estimates published by BLS. The estimated number of primary employment FTE workers is based on the BLS Current Population Survey, 2011.

§ Per 10,000 FTE workers. Rates were calculated by CDC based on the number of injuries and the number of primary employment FTE workers from the BLS Current Population Survey, 2011. CIs were calculated based on BLS-reported RSE where available. Variances were summed for collapsed industry and occupation categories. CDC calculated rates might differ from estimates published by BLS.

¶ DAFW cases include injuries that result in ≥1 days away from work with or without restricted work activity.

** Per 10,000 FTE workers. Each injury is only counted once, regardless of the number of ED visits. Rates were calculated by CDC based on the number of injuries and the number of primary employed FTE workers from the BLS Current Population Survey, 2011. Variances for NEISS-Work data and CPS data were pooled to estimate the variance for injury rates.

†† Data did not meet criteria for publication without compromise of confidentiality.

§§ Persons of Hispanic ethnicity might be of any race or combination of races.

¶¶ Rates were calculated based on 2011 County Business Patterns (information available at http://www.census.gov/econ/susb).

*** Industry in which the decedent worked was coded according to the 2007 NAICS (information available at http://www.census.gov/eos/www/naics). The detailed codes from the 20 NAICS sectors were combined into eight industry sectors according to the similarity of their occupational safety and health risks.

††† SOII industry counts, rates, and median DAFW were provided by BLS and are based on private industry only (excludes government employees).

§§§ Occupation in which the decedent worked was coded according to the 2010 SOC (available at http://www.blg.gov/soc). The detailed codes from the 22 civilian SOC groups were combined into 10 occupation groups according to the similarity of their occupational safety and health risks.

¶¶¶ Rates were calculated using all FTE workers as the denominator, based on the BLS Current Population Survey, 2011.

## Footnotes

* One full-time equivalent (FTE) worker = 2,000 hours worked per year.

† Analysis was conducted using restricted CFOI data that NIOSH receives through a memorandum of understanding. Results might differ from those released by BLS. To be considered an occupational fatality, the decedent of any age must have been employed at the time of the incident, working as a volunteer in the same capacity as a paid employee, or present at a site because of a job requirement.

§ BLS posts SOII data on their website for public use (http://www.bls.gov/iif/home.htm). As a collaborative federal/state survey program, SOII includes reports from a nationally representative sample of approximately 220,000 private-sector employers. Information about the survey methodology is available at http://www.bls.gov/opub/hom/homch9.pdf.

¶ NIOSH collects NEISS-Work data in collaboration with the Consumer Product Safety Commission (CPSC), which operates the base NEISS hospital system for the collection of data on consumer product–related injuries. NIOSH also collects the occupational injury data through collaboration with CPSC. However, there are no implied or expressed endorsements of the results presented herein by CPSC. The CPSC product-related injury estimates exclude work-related injuries, whereas NEISS-Work estimates include all work-related injuries regardless of product involvement (i.e., NEISS and NEISS-Work cases are mutually exclusive). From a stratified probability-based sample of 67 U.S. hospitals, injuries are determined to be work-related when the ED chart indicates an injury occurred to a noninstitutionalized civilian while working for pay or other compensation, working on a farm, or volunteering for an organized group.

** Occupational Injury and Illness Classification System version 2.01 was used to code cases obtained from CFOI and SOII. Version 1.01 was used to code cases obtained from NEISS-Work. Both versions are available at http://wwwn.cdc.gov/wisards/oiics.

†† CPS is the primary source of U.S. labor force statistics. The U.S. Census Bureau surveys approximately 50,000 households monthly to collect employment, unemployment, earnings, hours of work, and other indicators. Variances for CPS labor force estimates were calculated using the BLS approximate standard error formulas derived for CPS. The variances for NEISS-Work data and CPS data were pooled to estimate the variance for injury rates.
