# Erratum

**Published:** 2014-05-09

## Vol. 63, No. 16

In the report, “Occupational Ladder Fall Injuries — United States, 2011,” an error occurred in the first sentence of the first paragraph. The sentence reads, “Falls remain a leading cause of unintentional injury mortality nationwide, and 43% of fatal falls in the last decade have involved a ladder (*1*).” The last part of the sentence should be deleted, and the sentence should read, “Falls remain a leading cause of unintentional injury mortality nationwide (*1*).”

